# Trend and determinants of tobacco use among Indian males over a 22-year period (1998–2021) using nationally representative data

**DOI:** 10.1371/journal.pone.0308748

**Published:** 2024-10-22

**Authors:** Shibaji Gupta, Piyasa Mal, Dhiman Bhadra, Sathish Rajaa, Sonu Goel

**Affiliations:** 1 Department of Community Medicine, Midnapore Medical College, Medinipur, India; 2 Department of Public Health and Mortality Studies, International Institute for Population Sciences, Mumbai, India; 3 Operations and Decision Sciences Area, Indian Institute of Management Ahmedabad, Ahmedabad, India; 4 Department of Community Medicine, ESIC Medical College and PGIMSR, Chennai, India; 5 Department of Community Medicine and School of Public Health, Post Graduate Institute of Medical Education and Research, Chandigarh, India; 6 Public Health Masters Program, School of Medicine, University of Limerick, Limerick, Ireland; 7 Faculty of Human and Health Sciences, Swansea University, Wales, United Kingdom; ICMR - National Institute for Research in Reproductive and Child Health (ICMR-NIRRCH), INDIA

## Abstract

**Objective:**

Tobacco consumption is associated with an increased risk of morbidity and mortality. India is one of the largest consumers of tobacco worldwide. We assessed the trend of tobacco use among Indian males over a period of 20 years using data obtained from four rounds of the National Family Health Survey (NFHS).

**Methods:**

Data on tobacco usage and relevant socioeconomic variables obtained from NFHS rounds 2 to 5 over the period 1998–2021, was used for analysis. Specifically, data were available for 138,951 males from NFHS-2, 74,369 males from NFHS-3, 112,222 males from NFHS-4, and 101,839 males from NFHS-5. Significance of association between various socio-economic factors and tobacco usage was ascertained using a multicategory logistic regression model.

**Results:**

Among all the forms of tobacco, smokeless tobacco was predominantly used by Indian males. The proportion of smokers and those using both smoke and smokeless forms peaked during NFHS-3, followed by a consistent dip; however, the use of smokeless tobacco plateaued from NFHS-4 to NFHS-5. NFHS-5 shows that 19.2% of Indian males smoked, 27.0% used smokeless tobacco, and 6.3% used both. Tobacco use has declined significantly over the last two decades. Tobacco usage is noticeably higher among the elderly, Muslims, and those from the backward classes, while it was considerably lower for individuals belonging to the educated and wealthier segment.

**Conclusion:**

There has been a steady decline in tobacco use in India over the past 22 years, specifically in the smoke-form category. However, smokeless tobacco use remains nearly unaffected. The outcome of this study might aid policymakers in devising targeted tobacco control policies and improving existing ones.

## Introduction

The World Health Organization (WHO) lists tobacco use as one of the biggest public health threats, as it is a known risk factor for various chronic morbidities such as cardiovascular disease, stroke, lung ailments and cancer [1]. Besides adding to the mortality and morbidity tallies, tobacco use also incurs social and economic costs [1, 2]. Globally, India is the second largest producer and consumer of tobacco, only after China [1, 3]. Here, tobacco is most commonly used in its smokeless form, such as Khaini, Zarda, and Gutkha. Cigarettes, hookahs, and bidis are some of the smoke forms of tobacco which are commonly consumed [1].

The use of smokeless tobacco (SLT) lead to 2.5 million disability-adjusted life years (DALYs) and nearly 100,000 deaths globally due to oral, pharyngeal, and esophageal cancers in 2017. The highest prevalence of tobacco use in its smokeless form is found in South and South-east Asia [4]. Across the globe, smoke-form tobacco (SFT) was associated with 148.6 million DALYs and 7.1 million deaths due to various diseases in 2016. About 267 million Indians aged 15 years or above use tobacco [5]. Of total DALYs in India, 5.9% can be attributed to tobacco-use [4]. Tobacco-use accounts for 1.35 million deaths annually in India and results in huge social and economic costs for the country [1, 2].

India has a long history of implementing anti-tobacco measures. The Cinematograph Act of 1952, prohibits ‘glamorisation’ of smoking and tobacco in movies [6]. Since 2000, advertisement of tobacco products is banned in the country [7]. India passed the Cigarettes and other Tobacco Products Act (COTPA) in 2003, as a significant milestone in its effort against tobacco use and ratified WHO’s Framework Convention on Tobacco Control in 2005 [8, 9]. Currently, smoking in public places and the sale of tobacco products to minors or within 100-yards of educational institutions are banned. Cigarette, pan masala and other packets containing tobacco must mandatorily bear written and pictorial warnings [8, 9]. These measures have contributed to the decline in tobacco use in India over the years [10].

Both globally and in India, tobacco use of any form, is more commonly associated with males [10–13]. Almost 40% of Indian men (aged 15–54) consume tobacco, whereas for women (aged 15–49) it is 4% [4]. The economic burden due to tobacco-related diseases in India is majorly shared by males (91%) [14]. Hence, our paper specifically focuses on male tobacco consumption. Tobacco use is grossly under-reported in India and evidence indicates high chances of under-reporting of tobacco use specifically for females [15–17]. Numerous studies have shown a discrepancy between self-reported rates of tobacco use and those validated by biochemical analysis [18–21]. Thus including females would introduce additional variables and complexities, potentially diluting the study’s focus and leaving a scope of faulty estimates. By narrowing the scope to males, we can delve deeper into their unique trends and determinants, as this targeted approach allows for a more comprehensive analysis. This focused approach is intended to contribute valuable information for developing effective public health interventions and tobacco control policies tailored to the needs of male tobacco users.

The National Family Health Surveys (NFHS) have been providing nationally representative data for India since 1992–93 and have been generating valuable tobacco-use related data since 1998 [22]. Although there are some extent literature that assess trends of tobacco use in India, to the best of our knowledge, this is the first study that incorporates data from the latest round of NFHS, namely NFHS-5 This is probably because the final report of the same was released in May 2022 [23–25]. Hence, this study utilizes data from four rounds of NFHS, namely NFHS 2, 3, 4, and 5) to analyze the trend in tobacco use in India and assess the association of various socio-demographic variables with tobacco consumption. In doing so, we hope that our study will provide valuable insights regarding the impact of various government-sponsored anti-tobacco measures which have been implemented in India over the last 22 years.

### Objective

To analyze the trend in tobacco use among males in India and assess the association between various socio-economic and demographic factors and tobacco consumption using nationally representative data obtained from 4 round of National Family Health Surveys, namely NFHS 2 (1998–99), NFHS 3 (2005–06), NFHS 4 (2015–16) and NFHS 5 (2019–21).

## Methods

### Study design and participants

Data for this study are obtained from four rounds of the National Family Health Surveys, namely NFHS 2 (1998–99), NFHS 3 (2005–06), NFHS 3 (2015–16) and NFHS 5 (2019–21). Each of these surveys is designed to provide robust estimates of key indicators of health and family welfare like fertility, infant and child mortality, family planning practices, tobacco consumption, and various aspects of maternal and child health both at the national and sub-national levels. Data from successive rounds of NFHS are intended to aid policy makers in measuring the effectiveness of existing programs, setting benchmarks for new programs, and examining India’s progress towards meeting global targets such as the Sustainable Development Goals (SDG). All NFHS have been carried out under the stewardship of the Ministry of Health and Family Welfare (MoHFW), Government of India, with the International Institute of Population Sciences, Mumbai, acting as the nodal agency responsible for planning and execution.

NFHS-2 covered 91,196 households and 89,199 ever-married women aged 15–49 years, while NFHS-3 provided data on 124,385 women aged 15–49 years and 74,369 men aged 15–54 years. NFHS-4 covered 572,000 households, 699,686 women (aged 15–49) and 122,051 men (aged 15–54). The NFHS-5 was conducted from June 2019 to April 2021, with an interim gap due to COVID-19. It covered all 28 states, eight union territories, and 707 districts of India to arrive at reliable estimates of various health-related parameters. It included 101839 men aged 15–54 years and 724115 women aged 15–49 years having a response rate of 92% and 97% respectively [4]. The response rates for NFHS 2, 3, and 4 were 97.5%, 97.7%, and 97.6%, respectively.

Like other demographic health surveys, the NFHS follows a two-stage stratified-cluster sampling scheme to select a nationally representative sample of households from the entire country. In the first stage, each district was stratified into urban and rural zones, which were further sub-stratified into six similar-sized sub-strata based on multiple variables, such as the total population and concentration of Scheduled Castes (SC) and Scheduled tribes (ST)–underprivileged classes that have been specified by the Government of India. In the second stage, a sample of villages (rural areas) or Census Enumeration Blocks (urban areas) were selected as primary sampling units (PSU) from each stratum using proportional allocation. PSUs were sorted based on the women’s literacy rate (in rural areas) and the proportion of people belonging to backward classes (in urban areas). A PSU with 300 or more households was divided into smaller segments of 100–150 households. In the third stage, a random sample of 22 households was selected from each cluster (PSU or its segment) using an equal probability systematic random sampling scheme. Of the 30,456 (PSUs) selected across India, fieldwork was completed in 30,198 PSUs (S1 Fig). Men selected from a sub-sample of the selected households were interviewed using Man’s questionnaire after obtaining informed consent. The factors pertaining to this study that were included in the questionnaire were sociodemographic variables, media exposure, employment, and addiction, including tobacco use. Tobacco users were asked about the form(s) of tobacco they consumed.

The methodology of the survey has been discussed in greater detail in the recently published NFHS-5 report [4].

The sampled individuals have been categorized into the following 5 categories, based on their self-declaration:

1. Smokeless tobacco users: Current users of one or more smokeless tobacco substances (SLT), including paan with tobacco, khaini, snuff, gutkha/tobacco containing paan masala, and other forms of chewing tobacco.
2. Smoke-form tobacco users: Current users of one or more smoke-form tobacco substances (SFT), including cigarettes, bidi, cigar, pipes, and hookahs.
3. Both-form tobacco user: User of both forms of tobacco (BFT), i.e., SLT and SFT
4. Any-form tobacco users: Users of SLT, SFT, or both.
5. Non-users: Those who do not use any form of tobacco at present.

The independent variables were selected based on literature review, and included age (years), place of residence (urban/rural), district of stay, educational status, wealth status, marital status, religion, caste/tribe, work status, exposure to radio, and exposure to television. The reason for including radio and TV viewership is that exposure to mass media has a proven role in generating health awareness [26, 27]. The NFHS-5 report states that, “respondents were asked how often they read a newspaper or magazine, listened to the radio, watched television… Those who responded at least once a week… are considered to be regularly exposed to that form of media.” [4] Only those variables which were measured in all the aforementioned rounds of NFHS were included. The analysis-ready dataset only contained individuals for whom values for all the aforementioned variables were available.

### Statistical analysis

For each type of tobacco (smoke, smokeless, both), prevalence was defined as the number of persons consuming the given type of tobacco per 100 adults aged 15–54 years. We employed a multicategory logit model, specifically a baseline category logit model, to estimate and assess the adjusted associations between the different socio-economic and demographic characteristics and the prevalence of tobacco usage in each of the following categories: smoking form only, smokeless tobacco form only, dual use of tobacco, and non-users across the four survey rounds. The assumption behind using the baseline category logit model is that the aforementioned categories of users are unordered. In using the model, each of the first three categories was modelled relative to the baseline outcome group, namely non-users of tobacco. Accordingly, we report the relative risk ratios (RRR) along with the corresponding 95% confidence intervals. A relevant map of the country depicting burden of tobacco use was also created using ArcGIS 10.8 [28]. The survey protocols and questionnaires used by the NFHS were approved by the Institutional Review Board of the International Institute of Population Sciences (IIPS) in Mumbai. The United States Center of Disease Control and Prevention (CDC) also reviewed the protocol [4]. This study was approved by the Institutional Ethics Committee of a Medical College in India (Ref. No.: IEC-08/2022-2535 dated 17.8. 2022). Model fitting was carried out using Stata 16.0 (College Station, TX: StataCorp LLC).

## Results

### Trend of tobacco-use among men in India

**Fig 1:**
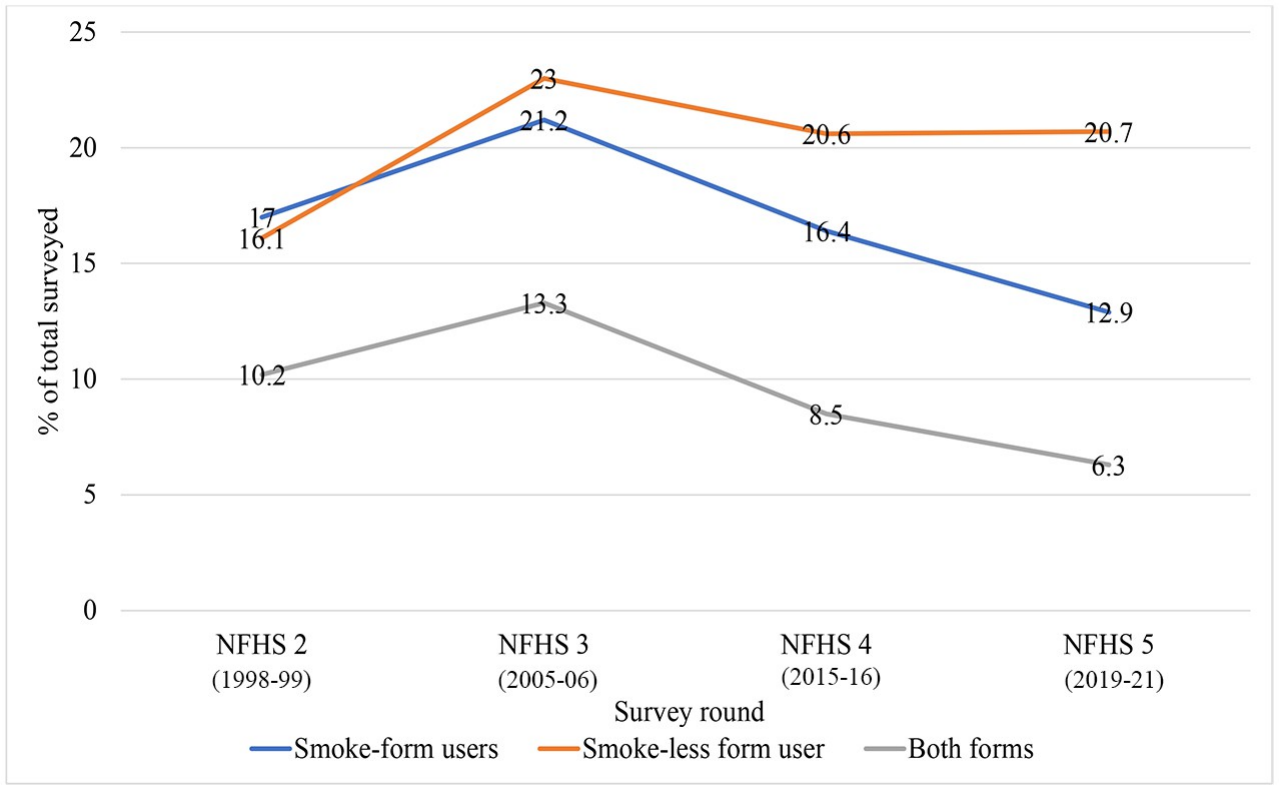
Trend of tobacco use among males across different rounds of NFHS.

We studied 138,951 males from NFHS-2, 74,369 from NFHS-3, and 112,222 and 101,839 men from NFHS-4 and 5 respectively (Table 1). Fig 1 shows the trend in tobacco use across the different rounds of NFHS. With an initial increase during 2005–06 (NFHS-3), SFT use showed a steady decline along with a decrease in BFT users. There was a decline in the proportion using SFT by 27.1% and BFT by 25.9% from NFHS-4 (2015–16). However, SLT remains the most common form of tobacco in India, with its consumption remaining nearly constant across NFHS 4 and 5. Khaini and chewing of tobacco containing paan-masala or gutkha have been the predominant form of SLT based on usage, while cigarettes are the most used SFT product. This trend was similar across all four rounds of NFHS (Table 2).

**Table 1:** Indian male tobacco users classified as per their socio-demographic profile.

	NFHS 2 (1998–99) (n = 138,951)			NFHS 3 (2005–06) (n = 74,369)			NFHS 4 (2015–16) (n = 112,122)			NFHS 5 (2019–21) (n = 101,839)		
	SLT	SFT	Any	SLT	SFT	Any	SLT	SFT	Any	SLT	SFT	Any
**Age**												
15–19	9.35	4.37	11.97	21.99	12.71	28.65	13.55	8.07	18.47	9.52	6.92	13.67
20–29	23.89	19.14	35.39	40.28	31.39	56.84	28.71	21.20	42.16	25.07	17.28	34.64
30–39	33.83	37.50	57.12	41.45	41.05	66.73	35.95	28.56	54.09	32.49	21.48	44.56
40–49	35.34	44.99	64.27	37.11	46.50	69.42	33.17	33.91	56.92	34.20	24.82	48.96
50–54	35.25	46.20	65.92	33.81	46.51	66.91	30.63	37.80	57.74	31.50	27.80	49.64
**Residence**												
Rural	28.90	30.32	47.45	39.44	37.32	61.79	32.78	23.14	49.16	30.83	19.94	41.81
Urban	20.13	19.79	33.64	30.97	29.73	50.26	23.16	26.01	39.66	19.93	17.91	31.52
**Education**												
No education	37.20	44.92	64.13	44.85	52.82	77.76	43.21	40.88	68.43	41.19	31.65	59.92
Incomplete primary	33.02	36.80	56.5	44.80	46.7	73.26	44.03	37.19	67.38	42.46	29.60	58.41
Incomplete secondary	25.04	24.77	40.76	36.04	30.69	54.39	30.67	24.12	46.21	26.64	17.37	36.51
Complete secondary	21.04	19.65	33.47	27.59	21.84	41.19	20.78	17.99	33.92	16.81	10.86	23.25
Higher	16.74	12.36	25.16	22.80	20.28	36.81	13.58	15.17	25.60	11.76	11.98	20.04
**Wealth**												
Poorest	40.21	38.33	60.06	50.81	45.88	74.84	48.94	31.68	64.18	44.41	26.72	57.80
Poorer	32.58	34.73	52.95	44.85	41.99	68.93	38.20	29.23	55.94	35.28	22.97	47.42
Middle	26.32	28.90	45.42	36.93	37.15	60.85	28.96	25.73	46.60	26.76	18.36	37.02
Richer	20.98	23.48	37.92	32.13	30.73	52.57	22.79	22.87	39.73	20.32	16.47	31.06
Richest	15.39	14.36	25.82	23.62	22.63	39.22	15.47	18.41	30	12.01	13.27	21.98
**Religion**												
Hinduism	27.26	27.39	44.17	37.10	34.80	58.16	30.01	24.75	46.08	27.70	18.59	38.31
Islam	23.79	29.72	43.69	35.76	37.87	61.09	27.17	27.73	46.67	26.16	21.97	39.53
Christianity	18.24	29.75	39.29	28.05	34.12	49.50	18.59	28.59	39.03	15.37	27.76	36.26
Other	19.92	12.26	27.16	25.58	16.52	35.65	20.67	14.07	30.24	22.46	13.37	28.70
**Caste/Tribe**												
SC	28.54	32.70	48.74	39.40	40.75	64.36	30.84	29.97	50.03	29.55	23.60	43.34
ST	39.74	32.28	56.96	50.06	40.96	71.66	42.38	27.35	57.70	38.09	21.81	50.15
OBC	26.03	25.99	41.72	35.54	32.89	55.33	28.33	23.13	43.52	26.06	15.63	34.69
Other	22.81	23.71	38.88	32.32	30.51	52.21	25.66	21.70	40.76	22.78	17.59	33.45
**Marital status**												
Unmarried	12.48	16.86	17.31	27.06	19.50	67.51	18.29	14.64	54.88	14.63	12.16	46.95
Currently married	33.83	33.92	57.53	40.91	42.16	81.32	34.85	30.25	69.96	33.73	22.93	62.75
Widowed/divorced/separated	38.76	38.97	67.28	52.01	50.78	37.82	43.13	44.10	27.90	43.25	34.51	21.99
**Work status**												
Non-Working	8.78	5.24	12.14	19.28	14.28	27.63	16.14	13.11	24.82	10.80	13.37	20.07
Working	30.48	32.43	50.80	39.33	38.10	62.81	33.09	28.55	51.91	21.86	31.25	43.86
**Exposure to radio**												
No	29.39	30.83	48.60	32.49	35.32	56.71	30.47	24.62	46.67	28.78	19.23	39.58
Yes	21.52	22.42	36.46	38.04	34.22	57.96	25.82	25.59	42.79	21.27	19.21	33.75
**Exposure to TV**												
No	31.31	32.73	50.77	43.45	42.64	70.10	43.14	28.68	58.95	38.53	22.23	50.28
Yes	18.42	18.50	31.69	34.82	32.81	54.88	26.91	24.32	43.43	24.37	18.54	35.45
**Total**	26.32	27.22	43.39	36.35	34.55	57.60	29.09	24.91	45.50	26.99	19.22	39.93

Numbers indicate % of total.

**Table 2:** Change in tobacco consumption among Indian men across survey rounds.

Tobacco Use	NFHS 2 (1998–99) (n = 138,951)	NFHS 3 (2005–06) (n = 74,369)	NFHS 4 (2015–16) (n = 112,122)	NFHS 5 (2019–21) (n = 101,839)
**SFT**				
Smokes cigarettes	27.2	33.4	13.7	13.3
Smokes bidis			14.3	7.8
Smokes cigars		0.7	0.5	0.6
Smokes a pipe			0.2	0.1
Smokes a hookah		NA	0.6	0.6
**SLT**				
Chews paan masala or gutkha	26.3	13.2	14.9	14.2
Uses khaini		23.6	12.4	12.1
Chews paan with tobacco			5.6	5.3
Other chewing tobacco			2.3	1.8
Uses snuff		0.7	0.1	0.1
Other		1.0	0.5	0.5
**Any form of tobacco**	43.4	57.6	45.5	39.9

Numbers indicate % of total; NA: Not Available

### Geographical variation in tobacco-use

**Fig 2:**
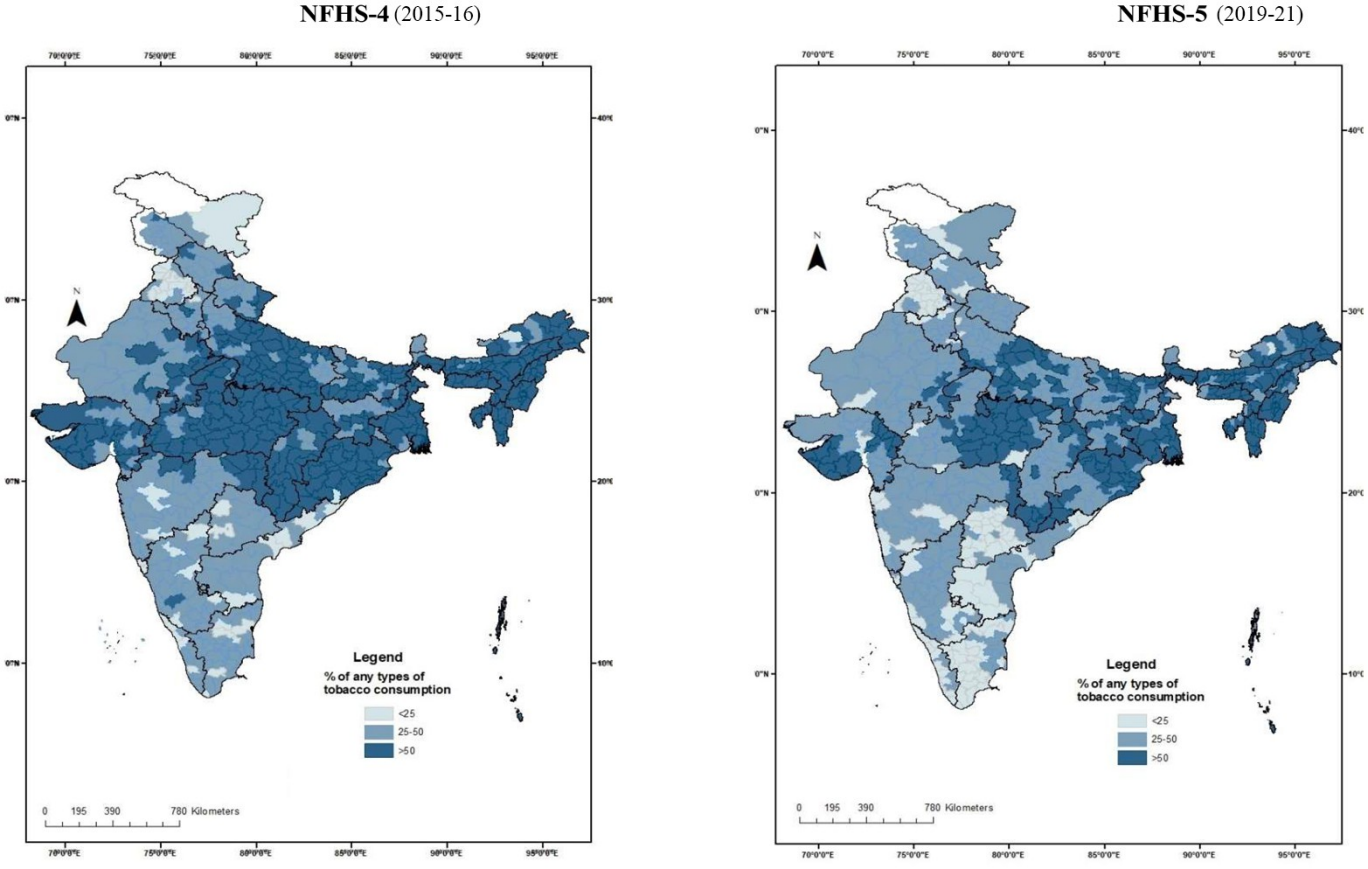
Burden of any-form tobacco use in districts of India among males according to NFHS 4 and 5^¶^. ^¶^created using ArcGIS 10.8. [21].

Fig 2 compares the district-wise burden of tobacco use (any form) in India between the NFHS-4 and 5 (2015–16 and 2019–21 respectively). The dark blue zones, indicating districts with more than 50% prevalence of tobacco use among participants, were found to have shrunken in NFHS-5when compared with NFHS-4. However, northeastern India continues to suffer from high tobacco use.

### Determinants of tobacco-use

#### Smoke-form tobacco (SFT)

As shown in Table 3, it is evident that with increasing age, the proportion of SFT users significantly increased, while the burden of SFT use was lower among the more educated and wealthier which is indicative of a negative association of education and wealth on tobacco consumption. After adjusting for other variables, urbanites tended to have a higher propensity to consume tobacco products. Interestingly, a significantly larger proportion of married participants tended to use tobacco compared to their unmarried counterparts, while widowed/divorced/separated participants reported less use of any form of tobacco in all rounds, except NFHS-2 (1998–99). Tobacco use seems to be related to religion and caste, with Muslims having a significantly higher propensity to use SFT compared to Hindus, while minorities other than Christians have a markedly lower propensity to use SFT compared to the Hindu majority. Participants from the general caste and other backward classes (OBCs) and scheduled tribes (STs) had a significantly lower chance of SFT use than those from the scheduled castes (SCs). On the other hand, the working class had a significantly higher usage of SFT than their non-working counterparts. Exposure to radio has a significant association with SFT usage based on NFHS-5 data, while the association is insignificant in the previous survey rounds. On the other hand, exposure to television had a significant association with tobacco consumption in all but the NFHS-5 sample. Overall, this is indicative of a time-varying association pattern between exposure to mass media and tobacco usage (Table 3).

**Table 3:** Association of socio-demographic variables with smoke-form tobacco-use (SFT) among Indian men across different rounds of NFHS.

	Relative Risk ratio (RRR) [95% confidence interval]			
	NFHS 2 (1998–99) (n = 138,951)	NFHS 3 (2005–06) (n = 74,369)	NFHS 4 (2015–16) (n = 112,122)	NFHS 5 (2019–21) (n = 101,839)
**Age**				
15–19	Ref	Ref	Ref	Ref
20–29	3.48***[3.11–3.89]	3.41***[3.00–3.89]	3.75***[3.28–4.27]	2.99***[2.46–3.62]
30–39	7.08***[6.27–7.98]	5.22***[4.49–6.07]	5.17***[4.43–6.04]	4.18***[3.35–5.21]
40–49	10.59***[9.36–11.98]	6.88***[5.89–8.03]	6.85***[5.85–8.04]	5.22***[4.18–6.52]
50–54	11.44***[10.00–13.09]	6.76***[5.68–8.05]	7.59***[6.40–9.01]	6.46***[5.10–8.20]
**Residence**				
Rural	Ref	Ref	Ref	Ref
Urban	0.95[0.90–1.00]	1.12**[1.04–1.20]	1.10**[1.02–1.18]	1.17**[1.05–1.29]
**Education**				
No education	Ref	Ref	Ref	Ref
Incomplete primary	0.85***[0.79–0.92]	0.83**[0.74–0.93]	0.93[0.82–1.06]	1.06[0.92–1.21]
Incomplete secondary	0.63***[0.59–0.66]	0.51***[0.47–0.56]	0.57***[0.52–0.62]	0.61***[0.54–0.68]
Complete secondary	0.39***[0.36–0.42]	0.31***[0.27–0.36]	0.42***[0.37–0.47]	0.51***[0.40–0.65]
Higher	0.26***[0.25–0.29]	0.26***[0.23–0.30]	0.31***[0.28–0.35]	0.36***[0.31–0.42]
**Wealth**				
Poorest	Ref	Ref	Ref	Ref
Poorer	1.06[0.99–1.14]	1.03[0.92–1.15]	1.01[0.92–1.11]	0.79***[0.69–0.89]
Middle	1[0.93–1.08]	0.88*[0.79–0.99]	0.83***[0.75–0.91]	0.57***[0.51–0.65]
Richer	0.93[0.85–1.01]	0.68***[0.60–0.76]	0.74***[0.66–0.82]	0.51***[0.45–0.59]
Richest	0.60***[0.54–0.67]	0.49***[0.43–0.56]	0.62***[0.55–0.70]	0.42***[0.36–0.51]
**Religion**				
Hinduism	Ref	Ref	Ref	Ref
Islam	1.17***[1.09–1.25]	1.23***[1.12–1.36]	1.19***[1.08–1.31]	1.26***[1.11–1.43]
Christianity	1.29***[1.14–1.44]	0.84*[0.71–0.99]	1.14[0.95–1.36]	1.62***[1.37–1.92]
Other	0.30***[0.26–0.35]	0.28***[0.23–0.33]	0.39***[0.32–0.47]	0.49***[0.35–0.69]
**Caste/Tribe**				
SC	Ref	Ref	Ref	Ref
ST	0.87***[0.80–0.94]	0.87*[0.77–0.99]	0.81***[0.74–0.90]	0.87*[0.77–0.99]
OBC	0.68***[0.65–0.72]	0.60***[0.55–0.65]	0.68***[0.63–0.73]	0.62***[0.56–0.69]
Other	0.87***[0.82–0.92]	0.76***[0.69–0.83]	0.74***[0.67–0.81]	0.85*[0.75–0.97]
**Marital status**				
Unmarried	Ref	Ref	Ref	Ref
Currently married	2.29***[2.14–2.46]	1.51**[1.17–1.94]	1.72***[1.40–2.12]	1.70***[1.28–2.25]
Widowed/divorced/separated	2.77***[2.38–3.23]	0.70***[0.64–0.77]	0.92[0.83–1.02]	0.95[0.83–1.09]
**Work status**				
Non-Working	Ref	Ref	Ref	Ref
Working	2.47***[2.25–2.71]	1.79***[1.59–2.01]	1.57***[1.44–1.71]	1.41***[1.24–1.60]
**Exposure to radio**				
No	Ref	Ref	Ref	Ref
Yes	0.98[0.93–1.02]	1.04[0.98–1.11]	1.01[0.95–1.08]	1.11*[1.01–1.22]
**Exposure to TV**				
No	Ref	Ref	Ref	Ref
Yes	0.93*[0.87–0.99]	1.13**[1.04–1.24]	1.21***[1.11–1.31]	1.12[1.00–1.27]

Each outcome was modelled relative to the baseline outcome group (non-users of tobacco).

* p<0.05,

** p<0.01,

*** p<0.001

#### Smokeless form of tobacco (SLT)

*As shown in* Table 4, age of the user had a positive association with SLT usage, while it had a negative association with education and wealth, that is, SLT use was lower among those who were better educated and wealthier. Region of residence was associated with SLT use, with higher usage among the urbanites accounting for other characteristics. While widowed/divorced/separated participants reported less usage of any kind of SLT in all rounds except NFHS-2 (1998–99), a significantly higher percentage of married participants tended to use SLT than unmarried participants. In most cases, individuals belonging to minority religions had a higher propensity to use SLT than Hindus did. Individuals belonging to the general caste and OBCs had a lower likelihood of using SLT than those belonging to the scheduled caste and tribes. Compared to the non-working class, the working class had a higher risk of SLT use. Because exposure to the media sometimes favored SLT use while other times did not, it can be said that the observed association varied over time. (Table 4).

**Table 4:** Association of socio-demographic variables with smoke-less tobacco-use (SLT) among Indian men across different rounds of NFHS.

	Relative Risk ratio (RRR) [95% confidence interval]			
	NFHS 2 (1998–99) (n = 138,951)	NFHS 3 (2005–06) (n = 74,369)	NFHS 4 (2015–16) (n = 112,122)	NFHS 5 (2019–21) (n = 101,839)
**Age**				
15–19	Ref	Ref	Ref	Ref
20–29	2.00***[1.85–2.16]	2.14***[1.93–2.37]	2.48***[2.26–2.72]	3.08***[2.73–3.48]
30–39	2.65***[2.42–2.90]	2.17***[1.92–2.47]	2.79***[2.50–3.12]	3.53***[3.05–4.08]
40–49	3.17***[2.89–3.49]	2.05***[1.79–2.34]	2.53***[2.25–2.84]	3.82***[3.29–4.44]
50–54	3.44***[3.08–3.85]	1.75***[1.48–2.07]	2.23***[1.94–2.56]	3.69***[3.11–4.39]
**Residence**				
Rural	Ref	Ref	Ref	Ref
Urban	1.13***[1.07–1.20]	1.17***[1.09–1.25]	1.11***[1.05–1.19]	1.03[0.95–1.13]
**Education**				
No education	Ref	Ref	Ref	Ref
Incomplete primary	1.06[0.99–1.14]	1.03[0.91–1.17]	1.16*[1.04–1.30]	1.27***[1.13–1.42]
Incomplete secondary	0.92**[0.87–0.97]	0.86**[0.79–0.94]	0.85***[0.79–0.92]	0.90*[0.82–0.99]
Complete secondary	0.70***[0.65–0.76]	0.62***[0.54–0.71]	0.61***[0.55–0.68]	0.80*[0.67–0.95]
Higher	0.67***[0.62–0.72]	0.48***[0.42–0.54]	0.38***[0.34–0.42]	0.39***[0.35–0.45]
**Wealth**				
Poorest	Ref	Ref	Ref	Ref
Poorer	0.78***[0.74–0.84]	0.85**[0.77–0.95]	0.72***[0.67–0.78]	0.69***[0.64–0.76]
Middle	0.65***[0.61–0.70]	0.63***[0.56–0.70]	0.47***[0.43–0.50]	0.44***[0.40–0.49]
Richer	0.52***[0.47–0.56]	0.49***[0.44–0.55]	0.35***[0.32–0.39]	0.33***[0.29–0.36]
Richest	0.33***[0.30–0.37]	0.34***[0.30–0.38]	0.24***[0.22–0.26]	0.20***[0.18–0.23]
**Religion**				
Hinduism	Ref	Ref	Ref	Ref
Islam	1.02[0.95–1.10]	1.12*[1.02–1.24]	1[0.92–1.08]	0.90*[0.82–0.99]
Christianity	0.53***[0.47–0.61]	0.52***[0.44–0.63]	0.47***[0.39–0.57]	0.42***[0.35–0.50]
Other	0.76***[0.68–0.84]	0.57***[0.50–0.65]	0.62***[0.54–0.72]	0.76*[0.63–0.92]
**Caste/Tribe**				
SC	Ref	Ref	Ref	Ref
ST	1.64***[1.52–1.77]	1.36***[1.21–1.54]	1.41***[1.30–1.53]	1.37***[1.24–1.52]
OBC	0.90***[0.85–0.95]	0.78***[0.72–0.85]	1[0.93–1.06]	1.04[0.96–1.12]
Other	1.02[0.97–1.09]	0.94[0.86–1.02]	1.16***[1.08–1.26]	1.13*[1.02–1.25]
**Marital status**				
Unmarried	Ref	Ref	Ref	Ref
Currently married	1.84***[1.73–1.96]	1.79***[1.37–2.33]	1.24*[1.01–1.52]	1.32*[1.04–1.68]
Widowed/divorced/separated	2.03***[1.73–2.37]	0.66***[0.60–0.72]	0.67***[0.62–0.73]	0.59***[0.53–0.65]
**Work status**				
Non-Working	Ref	Ref	Ref	Ref
Working	1.91***[1.78–2.06]	1.84***[1.66–2.03]	1.68***[1.57–1.80]	1.60***[1.47–1.75]
**Exposure to radio**				
No	Ref	Ref	Ref	Ref
Yes	0.95*[0.91–0.99]	1.33***[1.25–1.41]	0.84***[0.80–0.89]	0.81***[0.75–0.88]
**Exposure to TV**				
No	Ref	Ref	Ref	Ref
Yes	1.02[0.96–1.09	0.95[0.87–1.04]	0.93*[0.87–1.00]	0.82***[0.77–0.89]

Each outcome was modelled relative to the baseline outcome group (non-users of tobacco).

* p<0.05,

** p<0.01,

*** p<0.001

#### Both-form tobacco (BFT)

As shown in Table 5, age was positively associated with BFT usage. Like SFT and SLT, the propensity for BFT use was lower for individuals who are better educated and belong to higher wealth categories. Controlling for other factors, individuals hailing from urban zones were at higher risk of BFT use. In all rounds, except for NFHS-2, people who were widowed, divorced, or separated used BFT less often. However, married participants were considerably more likely to use the BFT than unmarried participants. Muslims were more likely to use BFT, while people from the general caste and OBC were less likely to use it compared to those from the scheduled castes and tribes. As was true for SFT and SLT use, the association of mass media with BFT use cannot be ascertained with confidence.

**Table 5:** Association of socio-demographic variables with both-form tobacco-use among Indian men across different rounds of NFHS.

	Relative Risk ratio (RRR) [95% confidence interval]			
	NFHS 2 (1998–99) (n = 138,951)	NFHS 3 (2005–06) (n = 74,369)	NFHS 4 (2015–16) (n = 112,122)	NFHS 5 (2019–21) (n = 101,839)
**Age**				
15–19	Ref	Ref	Ref	Ref
20–29	3.32***[2.91–3.78]	3.37***[2.92–3.89]	3.67***[3.15–4.27]	3.57***[2.86–4.45]
30–39	5.98***[5.19–6.89]	3.62***[3.05–4.29]	4.45***[3.74–5.30]	3.75***[2.93–4.81]
40–49	8.35***[7.22–9.65]	3.39***[2.84–4.06]	4.35***[3.65–5.20]	3.95***[3.06–5.11]
50–54	8.46***[7.22–9.92]	3.10***[2.51–3.83]	4.50***[3.70–5.49]	3.65***[2.75–4.85]
**Residence**				
Rural	Ref	Ref	Ref	Ref
Urban	1.10**[1.03–1.18]	1.22***[1.12–1.33]	1.25***[1.15–1.37]	1.38***[1.21–1.57]
**Education**				
No education	Ref	Ref	Ref	Ref
Incomplete primary	0.83***[0.77–0.90]	0.9[0.79–1.03]	0.97[0.85–1.11]	1.08[0.92–1.28]
Incomplete secondary	0.71***[0.67–0.75]	0.62***[0.56–0.68]	0.63***[0.58–0.69]	0.61***[0.53–0.71]
Complete secondary	0.54***[0.50–0.59]	0.36***[0.30–0.43]	0.37***[0.32–0.42]	0.41***[0.30–0.57]
Higher	0.34***[0.31–0.37]	0.25***[0.21–0.30]	0.22***[0.19–0.26]	0.30***[0.24–0.38]
**Wealth**				
Poorest	Ref	Ref	Ref	Ref
Poorer	0.78***[0.73–0.84]	0.76***[0.68–0.86]	0.61***[0.56–0.66]	0.63***[0.55–0.71]
Middle	0.52***[0.48–0.56]	0.48***[0.42–0.54]	0.36***[0.32–0.40]	0.33***[0.29–0.39]
Richer	0.36***[0.32–0.40]	0.33***[0.29–0.38]	0.24***[0.22–0.27]	0.24***[0.20–0.28]
Richest	0.21***[0.18–0.25]	0.22***[0.19–0.26]	0.17***[0.14–0.20]	0.12***[0.09–0.16]
**Religion**				
Hinduism	Ref	Ref	Ref	Ref
Islam	1.20***[1.10–1.30]	1.01[0.89–1.14]	1.07[0.95–1.20]	0.88[0.74–1.05]
Christianity	0.91[0.80–1.04]	0.85[0.72–1.02]	0.96[0.80–1.16]	0.87[0.73–1.04]
Other	0.46***[0.38–0.54]	0.33***[0.26–0.40]	0.41***[0.33–0.49]	0.71**[0.56–0.91]
**Caste/Tribe**				
SC	Ref	Ref	Ref	Ref
ST	1.15**[1.06–1.25]	1.15*[1.00–1.32]	0.95[0.86–1.05]	0.89[0.77–1.03]
OBC	0.79***[0.74–0.84]	0.71***[0.65–0.78]	0.74***[0.68–0.80]	0.70***[0.62–0.79]
Other	0.80***[0.74–0.85]	0.81***[0.73–0.90]	0.81***[0.72–0.90]	0.82*[0.69–0.97]
**Marital status**				
Unmarried	Ref	Ref	Ref	Ref
Currently married	2.52***[2.31–2.75]	1.87***[1.41–2.50]	1.98***[1.57–2.49]	1.99***[1.49–2.65]
Widowed/divorced/separated	3.06***[2.56–3.66]	0.68***[0.61–0.76]	0.81***[0.73–0.91]	0.84*[0.72–0.98]
**Work status**				
Non-Working	Ref	Ref	Ref	Ref
Working	2.40***[2.14–2.69]	1.89***[1.63–2.18]	1.71***[1.55–1.88]	1.76***[1.53–2.04]
**Exposure to radio**				
No	Ref	Ref	Ref	Ref
Yes	1.02[0.96–1.08]	1.56***[1.44–1.70]	1.11**[1.04–1.20]	0.97[0.86–1.09]
**Exposure to TV**				
No	Ref	Ref	Ref	Ref
Yes	0.87**[0.80–0.95]	1.19**[1.07–1.32]	1.07[0.98–1.16]	0.97[0.87–1.09]

Each outcome was modelled relative to the baseline outcome group (non-users of tobacco).

* p<0.05,

** p<0.01,

*** p<0.001

## Discussion

This study assessed the trends and determinants of tobacco use among Indian males over a period of 22 years using nationally representative data obtained from four rounds of the National Family Health Survey (NFHS). The NFHS provides reliable estimates of various health indicators across India and strengthens countries’ demographic and health databases. The NFHS has provided tobacco-related data since 1998 (Round 2).

### Trend of tobacco use

The results obtained from our analysis clearly show a declining trend in tobacco consumption in India, a fact corroborated by Global Adult Tobacco Survey (GATS) and WHO reports [5, 10]. In 2004, India ratified the WHO-Framework Convention on Tobacco Control (WHO-FCTC) [29]. The declining trend in tobacco usage since 2005–06 (NFHS-3) could be the result of adoption of this international treaty, which seeks to tackle some of the factors associated with tobacco-use such as tobacco advertising, promotion, and illicit trade [29]. Higher price of tobacco products is an influential strategy for decreasing tobacco usage as well [30–34]. Average prices of cigarettes increased three-folds, while those of bidis and SLTs increased two-folds, between 2009 and 2016–17 [35]. NFHS-5 data shows a prominent decline in tobacco smokers and both-form users. District-wise tobacco use declined between the last two surveys (NFHS 4 and 5). However, smokeless form use remained stable across the survey waves (+0.5% change). The prevalence of tobacco use continues to remain high in some districts, especially in eastern and northeastern India.

### Factors associated with tobacco use

Age is an important determinant of tobacco use, with the elderly being more prone to tobacco use than the younger generation. This is a well-accepted fact in the extant literature [12, 36, 37]. A similar trend has been seen in South-east Asian countries as well [38]. Though tobacco use among the young have shown a decline [5], loosening societal constraints has led to easy availability of tobacco products [35]. Chance of under-reporting due to fear of reprimand and social stigma cannot be ruled out [37]. Despite having lower usage, the propensity for tobacco usage was higher among those hailing from urban localities compared to their rural counterparts.

Tobacco has deep-seated roots in Indian culture and is related to social status and cultural identity. Offering and sharing tobacco products, especially SFLT, is a widely accepted means of social bonding. Apart from their strong association with culture, lower costs and ease of availability are also vital factors behind the observed stability of SFLT use [20, 29, 35]. Social factors and traditions could also explain the significant differences in tobacco use across various castes and tribes in India. Having said that, the scope of under-reporting remains.

Education and wealth have a significant negative association with prevalence of tobacco use, a fact that finds ample corroboration in the extent literature [37–41]. This can be attributed to lesser awareness of the harmful effects of tobacco products among the lesser educated and poorer classes possibly stemming from inadequate access to schooling as well as to digital and mass media [35]. Individuals with better education have been shown to be less likely to be smokers [42]. On the other hand, we find a significantly higher prevalence of tobacco use among the employed class compared to the non-working section of the populace. This fact is also corroborated in the existing literature [42, 43]. The divergent association patterns of education and employment with tobacco usage may seem to be counterintuitive, but it must be remembered that a significant proportion of employment in India is in the informal sector which primarily employs people from lower socio-economic strata, that is, belonging to the lesser wealth and education category. Propensity to use tobacco products, especially cheaper ones, is significantly higher in this group. Moreover, those with higher income and education in the employed segment are more prone to consume cigarettes and costlier tobacco products. Inequitable access to existing strategies and the effectiveness of different programmatic strategies may also be important; however, the same could not be verified with NFHS data, as no awareness-related questions were asked. The association between mass media and tobacco usage had varying patterns across the different rounds of NFHS. Therefore, media power requires careful use in the public interest.

### The way forward

It is evident that although a significant reduction in tobacco usage and consumption has been achieved, there is a lot more left to do. For instance, SLT usage has not shown a significant decline, and some parts of India still have a high prevalence of tobacco use. In addition, disadvantaged sections are more prone to tobacco-related behavior. Hence, in continuation with the efforts already in place, targeted region-specific policies and interventions need to be framed specifically for the most susceptible population subgroups. Regular awareness campaigns involving local influencers and better use of social and mass media can be effective in this space. Media campaigns can enhance awareness of tobacco-related health hazards and anti-tobacco campaigns owing to their higher penetration among the masses. In fact, mass media campaigns, television, and radio advertisements, have been found to have a positive impact towards smoking cessation and intention to quit. as well as being comparatively cost-effective [26, 27]. Towards this end, interactive social media applications can be developed to harness the power of Artificial Intelligence, and a stronger network of counsellors with an active tracking system for people willing to quit may be implemented. The Government has already implemented the ‘mCessation’ initiative based on text messaging to help those willing to quit. Toll-free helplines have been established to encourage tobacco-users and addicts wishing to quit to reach out for help and rehabilitation [44]. Children and young adults who are prone to secondhand smoke and are at risk of tobacco initiation can be sensitized through planned curricula and counselling sessions at schools and colleges. Tobacco-free educational campuses, as mandated by law, also need to be guaranteed [9]. Ground level health workers like the Accredited Social Health Activists (ASHAs) and Anganwadi workers can be asked to counsel members of households who are active tobacco consumers.

Tobacco product-related promotional activities often escape existing laws through loopholes. Surrogate advertisements for tobacco products are rampant in print media and television, and point-of-sale advertising creates common visuals. Therefore, the existing laws also require modifications. The 6 MPOWER measures, as laid down by WHO, can be implemented to strengthen existing measures [45]. This includes, monitoring of tobacco use and prevention policies, protecting people from tobacco use, offering help to quit tobacco use, warning about the dangers of tobacco, enforcing bans on tobacco advertising, promotion and sponsorship and raising taxes on tobacco [45]. A hotline for the public to report violations of laws can be useful. Further hikes in tobacco taxes on manufacturing, distribution, and usage might also be needed. Reducing tobacco cultivation and providing alternative employment to tobacco-cultivators is needed to eradicate the problem at its roots [35].

### Limitations and strengths

The findings relied on self-reported data from the NFHS, which may be prone to social desirability bias. Chances of recall bias cannot be eliminated. The level of tobacco-related awareness could not be determined because of the lack of available data. Gender-specific patterns could not be assessed because of the exclusion of women from the analysis.

Our analysis was derived from a large and nationally representative dataset, making the findings generalizable to Indian men. This study covers a 22-year period for its repeated cross-sectional analysis using a multinomial logit model for robust statistical analysis. This study provides rich insights into tobacco use patterns and determinants as well as capturing the latest trends in tobacco use among Indians. This could be useful for policymakers in designing effective interventions.

## Conclusion

India has been implementing anti-tobacco policies for several decades with Initiatives like The National Tobacco Control Program of India and the COTPA (Cigarettes and other Tobacco Products Act) that restricts tobacco sale and use.

Despite serious efforts, tobacco use is still prevalent in India, especially among disadvantaged groups. So, it is important to evaluate the effectiveness of the existing anti-tobacco policies separately in different cultural and socio-economic groups and redesign them as per needs [37]. Banning tobacco would mean huge immediate financial losses for the government, stalling economic growth, and a significant loss of employment.—Therefore, a well-thought multi-faceted approach needs to be adopted, mainly targeted at increasing public awareness and repulsion towards tobacco products.

## Supporting information

S1 Fig NFHS-5 sampling technique. (TIF)
