# Supplementary figures and images for: Trend and determinants of tobacco use among Indian males over a 22-year period (1998–2021) using nationally representative data
Source: PLoS One. 2024 Oct 22;19(10):e0308748. doi: 10.1371/journal.pone.0308748 (PMC11495595; doi:10.1371/journal.pone.0308748)

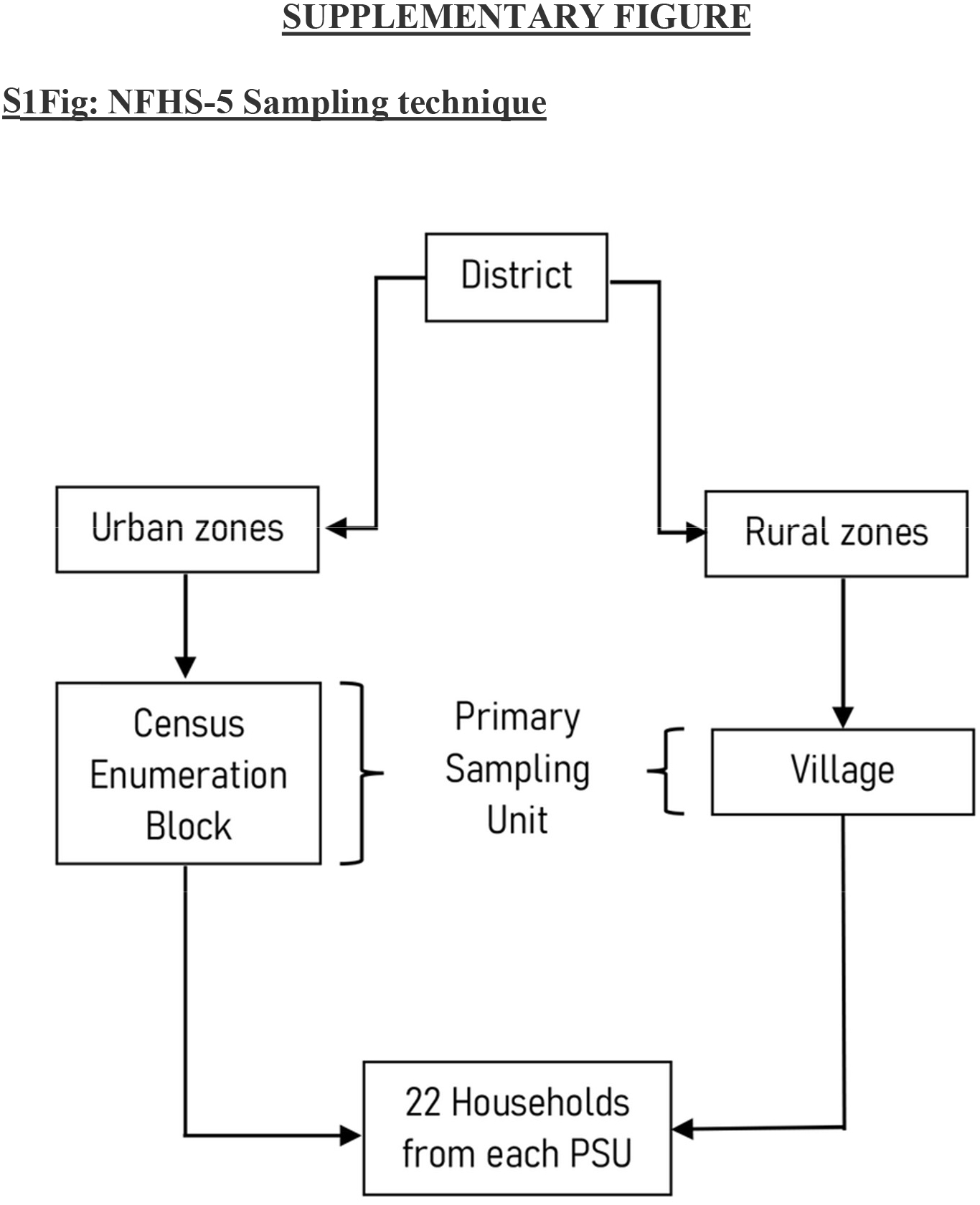

Supplement: S1 Fig — (TIF) [file pone.0308748.s001.tif]
